# Computational insights into the mechanisms underlying structural destabilization and recovery in trafficking-deficient hERG mutants

**DOI:** 10.3389/fmolb.2024.1341727

**Published:** 2024-08-13

**Authors:** Sara AlRawashdeh, Farag E. S. Mosa, Khaled H. Barakat

**Affiliations:** Faculty of Pharmacy and Pharmaceutical Sciences, University of Alberta, Edmonton, AB, Canada

**Keywords:** hERG potassium ion channel, hereditary LQTS, LQT2, intracellular retention, structural destabilization, trafficking rescue, AMD simulations

## Abstract

Cardiovascular diseases are a major global health concern, responsible for a significant number of deaths each year, often linked to cardiac arrhythmias resulting from dysfunction in ion channels. Hereditary Long QT Syndrome (LQTS) is a condition characterized by a prolonged QT interval on ECG, increasing the risk of sudden cardiac death. The most common type of LQTS, LQT2, is caused by mutations in the hERG gene, affecting a potassium ion channel. The majority of these mutations disrupt the channel’s trafficking to the cell membrane, leading to intracellular retention. Specific high-affinity hERG blockers (e.g., E-4031) can rescue this mutant phenotype, but the exact mechanism is unknown. This study used accelerated molecular dynamics simulations to investigate how these mutations affect the hERG channel’s structure, folding, endoplasmic reticulum (ER) retention, and trafficking. We reveal that these mutations induce structural changes in the channel, narrowing its central pore and altering the conformation of the intracellular domains. These changes expose internalization signals that contribute to ER retention and degradation of the mutant hERG channels. Moreover, the study found that the trafficking rescue drug E-4031 can inhibit these structural changes, potentially rescuing the mutant channels. This research offers valuable insights into the structural issues responsible for the degradation of rescuable transmembrane trafficking mutants. Understanding the defective trafficking structure of the hERG channel could help identify binding sites for small molecules capable of restoring proper folding and facilitating channel trafficking. This knowledge has the potential to lead to mechanism-based therapies that address the condition at the cellular level, which may prove more effective than treating clinical symptoms, ultimately offering hope for individuals with hereditary Long QT Syndrome.

## 1 Introduction

**FIGURE 1:**
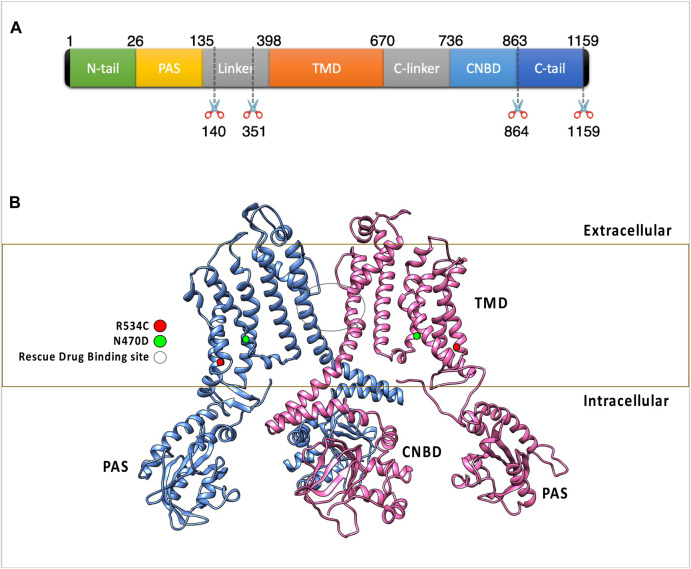
**(A)** The figure highlights the subdomain structure of the hERG channel monomer. Notably, the AlphaFold model of the hERG monomer excludes two disordered regions from the full-length sequence. **(B)** The arrangement of two opposing subunits in the hERG channel, emphasizing the binding site of the trafficking rescue drugs and the positions of two transmembrane mutations. These two mutations were classified as trafficking-defective hERG mutants during experimental assessments and were experimentally found to be rescuable by the trafficking rescue drug E-4031, R534C (Mesquita et al., 2019), and N470D (Gong et al., 2004) as well as the binding site of the trafficking rescue drugs.

The human ether-a-go-go related gene (hERG or KCNH2) encodes the pore-forming subunit of the Kv11.1 voltage-gated potassium ion channel. hERG plays a key role in ventricular repolarization, essential for the contraction of cardiac cells (Zhou et al., 1998; Zequn and Jiangfang, 2021). Loss of hERG function is associated with prolongation of the QT interval, leading to a high-risk condition known as long-QT syndrome type-2 (LQT2) (Anderson et al., 2006). Compromised repolarization, prolonged cardiac action potential and elevated risk of sudden death, seizures, and fatal torsades de pointes arrhythmia are characteristics of LQT2 (Anderson et al., 2006). In terms of structure, the hERG channel subunits assemble into a tetramer comprising a transmembrane region (TMD) and two intracellular structures. The cystolic domains of the hERG channel includes the N-terminal Per-Arnt-Sim (PAS) domain and the C-terminal cyclic nucleotide-binding domain (CNBD) (Figure 1) (Wang and MacKinnon, 2017; Butler et al., 2020; Robertson and Morais-Cabral, 2020; Asai et al., 2021). The transmembrane domain of the tetramer forms the channel cavity and includes the voltage sensor, the channel selectivity filter, and the ion-conducting pore (Wang and MacKinnon, 2017; Butler et al., 2020; Robertson and Morais-Cabral, 2020; Asai et al., 2021).

Defects in the hERG gene cause subunit abnormalities in inherited LQT2 syndrome, the most prevalent kind of hereditary LQTS. Single amino acid mutations are the primary cause of this condition, with roughly 200 mutations fully characterized with functional studies so far (Al-Moubarak et al., 2020; Oliveira-Mendes et al., 2021). In 90% of these mutations, the loss of function of hERG channels is mediated by faulty protein intracellular transport and trafficking to the cell membrane (hereditary LQT2 type 2) (Foo et al., 2016). Other causes of hERG function loss in these mutations include faulty channel synthesis, abnormal channel gating, or permeation. All these defects are thought to be due to a protein conformational issue (Thomas et al., 2003; Anderson et al., 2006). Similar to other membrane proteins, newly formed wildtype hERG channels are synthesized, folded, assembled, and N-linked glycosylated in the endoplasmic reticulum (ER) in the same manner as membrane proteins are (Orellana, 2019). Properly folded and assembled channels are transported to the Golgi, where they mature via glycan modification. This maturation increases the protein’s molecular mass from 135 kDa (in its immature form) to 155 kDa (in its mature form). Mature channels are subsequently directed to their final destination at the cell membrane (Napp et al., 2005; Dennis et al., 2007; Orellana, 2019; Wu et al., 2019).

An increasing number of human disorders have been associated with faulty protein trafficking, believed to result from improper protein folding and/or incorrect molecular assembly. Correcting protein trafficking abnormalities has become a therapeutic goal in several diseases, including cystic fibrosis (Carlile et al., 2007) and nephrogenic diabetes insipidus (Carlile et al., 2007). This is mainly because many of these trafficking- defective membrane proteins can still perform their intended functions when they are induced to correct their folding state (Luo et al., 2021). For example, when tested at a physiological temperature, hERG mutants in hereditary LQT2 type 2 were shown to be trafficking defective. Culturing mutant-expressing cells at 37°C resulted in the formation of only immature channel protein, which was held intracellularly and failed to travel to the plasma membrane (Kupershmidt et al., 2002; Thomas et al., 2003; Yeung and Meanwell, 2008; Nogawa and Kawai, 2014; Mura et al., 2017; Zheng et al., 2022). When the same cell line was cultured at a lower temperature (27°C), trafficking of the mutant channel protein into the plasma membrane was restored, as did the production of hERG current. It was also reported that cultivating the mutant cell line at 37°C in the presence of high-affinity hERG channel-blocking compounds such as E-4031 dihydrochloride, cisapride and astemizole restored the synthesis of the mature protein (Zhou et al., 1998; Kupershmidt et al., 2002; Anderson et al., 2014). After removing the drug, hERG current could be measured, demonstrating the trafficking of functional channels into the plasma membrane (Thomas et al., 2003; Yeung and Meanwell, 2008). These investigations demonstrated that mutant hERG channel proteins may be pharmacologically restored, raising the possibility of a novel therapeutic method (Anderson et al., 2014). Nonetheless, pharmacological rescue with high-affinity hERG channel-blocking medicines occurred at concentrations that resulted in total block, making this method look less promising since the mechanism of hERG channel drug block and pharmacological rescue appeared to be inexorably intertwined. LQT2 linked trafficking mutations are hypothesised to cause conformational abnormalities in the hERG channel, resulting in recognition and degradation by unique protein quality control machinery at the endoplasmic reticulum (ER). By binding to a location inside the channel pore, these known hERG “pharmacochaperones” may rectify the mutant conformational defect and increase hERG trafficking. In light of this, numerous innovative techniques have arisen. This includes transcriptional regulators (Thomas et al., 2003), RNA interference and pharmacological chaperones (Thomas et al., 2003; Gong et al., 2004; Matsa et al., 2014), with pharmacological rescue of trafficking-deficient hERG channel proteins seeming to be the most promising strategy as yet (Rajamani et al., 2002; Luo et al., 2021).

The mechanisms underlying the restoration of hERG channel trafficking remain unclear. The first human hERG channel experimental structure has been released as a result of recent advances in cryo-EM techniques (PDB (Berman et al., 2000) ID: 5VA2 (Wang and MacKinnon, 2017)). In 2021, another cryo-EM structure of the human hERG channel was published (PDB ID: 7CN1 (Asai et al., 2021)). This breakthrough is regarded as the foundation for the application of computational techniques to the study of hERG mutants and their pharmacological restoration. Computational methods are well suited to improving the efficiency of experimental techniques in terms of investigating those systems in atomistic detail and analysing the structure and dynamics of these mutants under physiological conditions. Computational simulation is an efficient tool for studying these systems and investigating their structural destabilization and mechanism of pharmacological rescue (Hertig et al., 2016; Ghattas et al., 2018; Duan et al., 2019; Ghattas et al., 2020; Jin et al., 2021).

Understanding hERG folding, assembly, and ER export processes can be improved by studying the dynamics of these mutants and the effects of small molecules on their stability. Furthermore, studying the folding state and dynamics of wildtype and trafficking-deficient mutant channels can help reveal key structural differences that lead to mutant channel trafficking failure. Finally, studying the trafficking defective structure of the hERG channel can provide information on binding sites for small molecules that can restore the channel’s functional folding state, thus restoring channel trafficking.

In this work, we used accelerated molecular dynamics simulations (Hamelberg et al., 2004; Grant et al., 2009; Bucher et al., 2011; de Oliveira et al., 2011; Mücksch and Urbassek, 2013) to investigate the effect of rescuable transmembrane domain (TMD) trafficking mutations N470D (Gong et al., 2004) and R534C (Mesquita et al., 2019) (Figure 1) on the hERG protein structure. The current study sheds light on the mechanism by which structural destabilization can lead to ER retention in hERG mutants and explores how this effect can be reversed when the hERG trafficking rescue drug E-4031 binds to these trafficking mutants.

## 2 Computational methods

For the hERG model we tetramerized and core glycosylated the AlphaFold (Jumper et al., 2021) generated monomer of the hERG protein. The AlphaFold monomer sequence lacks two disordered portions from the full-length monomer (Figure 1), however those missing segments were found to have no effect on channel trafficking or conductance function at the cell membrane (Wang and MacKinnon, 2017).

### 2.1 Building wildtype and mutant simulated systems

The hERG wildtype monomer employed in this study was sourced from the AlphaFold (Jumper et al., 2021) Protein Structure Database (http://alphafold.ebi.ac.uk, AlphaFold ID AF-Q12809-F1). Following this, segments characterized by low model confidence and lack of structure were excluded (see Figure 1). AlphaFold provides a confidence score for each residue, determined by the pLDDT (predicted local-distance difference test). The pLDDT score spans from 1 to 100, where 1 indicates low confidence and 100 signifies high confidence. The preference for using the AlphaFold model over two available cryo-EM structures (Wang and MacKinnon, 2017; Asai et al., 2021) for the hERG protein arises due to the inadequacies of the existing cryo-EM structures. These structures either lack complete hERG domains or exhibit substantial omissions in terms of domains or domain linkers. Notably, the AlphaFold model incorporates the existing cryo-EM structure and provides predictions for the missing segments, offering a more comprehensive representation. The full hERG channel tetramer was constructed using Phenix (Liebschner et al., 2019) which used the REMARK 350 lines of the hERG channel PDB ID: 5VA1 (Wang and MacKinnon, 2017). These lines include the transformations (rotational and translational), both crystallographic and non-crystallographic, required to generate the biological tetramer from the deposited coordinates of the monomer. The core N-glycan Glc3Man9GlcNAc2 (Napp et al., 2005) was built and linked to asparagine 598 (Napp et al., 2005) of all monomers using the GlycanModeler (Park et al., 2019). Mutations for the mutant systems were generated using the ResidueScan (Molecular Operating Environment MOE, 2022) module of the MOE package (Molecular Operating Environment MOE, 2022). For the drug-bound mutants’ systems, the trafficking rescue drug E-4031 was docked into the channel tetramer pore using the GLIDE module (Friesner et al., 2004) of the Maestro package (Schrödinger Release, 2021). During the docking process, the Glide grid (Friesner et al., 2004) was generated using the previously identified pore-binding site residues (Ficker et al., 2002; Butler et al., 2020; Robertson and Morais-Cabral, 2020), and all default Glide (Friesner et al., 2004) docking parameters were employed. The choice of the docked pose for subsequent MD simulations relied on selecting the pose with the best docking score, while also ensuring interactions with the previously identified hERG pore binding site residues—Tyr652, Ser624, and Thr623 (Robertson and Morais-Cabral, 2020)—during visual inspection of the poses. The docking scores for the chosen poses are detailed in Supplementary Table S3.

The construction of the ER membrane encasing the glycosylated tetramer was carried out using the CHARMM-GUI (Jo et al., 2008; Wu et al., 2014) server. The composition of lipids selected for the simulated setups represented the most realistic mammalian ER membrane (Pogozheva et al., 2022) and is detailed in Supplementary Table S1, summarizing their relative ratios. On average, each leaflet in each bilayer system consisted of approximately 250 lipids. It is important to highlight that all systems investigated in this research exhibited remarkable intricacy, encompassing as many as 12 distinct lipid varieties. The membrane assembly was symmetrical in all cases and all systems were generated by MembraneBuilder (Jo et al., 2009; Wu et al., 2014; Lee et al., 2019). Force field parameters of the systems and ligand were taken from the CHARMM36 (Huang and MacKerell Jr, 2013; Croitoru et al., 2021) force field then converted to AMBER (Wang et al., 2004; Salomon-Ferrer et al., 2013) input files in the final step of the CHARMM-GUI (Jo et al., 2008) server’s protocol. In the CHARMM-GUI protocol, the parameterization of all system components, encompassing protein, lipid, and ligand, is carried out using the CHARM36 force field. Users are provided with the flexibility to convert these parameterizations to AMBER force fields, with Lipid14 (Dickson et al., 2014) for lipids, ff19SB (Tian et al., 2020) for proteins, GLYCAM_06j (Kirschner et al., 2008) for glycans, and gaff2 (He et al., 2020) for ligands. For both classical and accelerated molecular dynamics simulations, the AMBER20 package (Salomon-Ferrer et al., 2013) was employed as the simulation engine with inputs generated by CHARMM-GUI (Jo et al., 2008) where water molecules, ions (150 mM NaCl) and counter Ca^2+^ ions to neutralize the systems were added.

### 2.2 Classical molecular dynamics simulations

We followed the standard six-step equilibration procedure outlined by MembraneBuilder (Jo et al., 2009). These steps include reading the protein structure, adjusting protein orientation if necessary, determining the system size, constructing bilayer components, assembling these components into a bilayer, and finally, equilibrating the entire system. We then initiated an NVT (constant particle number, volume, and temperature) molecular dynamics simulation for each system. This used a 1 femtosecond (fs) time step for a duration of 2 nanoseconds (ns). Subsequently, we transitioned to an NPT (constant particle number, pressure, and temperature) ensemble, employing a 1 fs time step for 2 ns and then shifting to a 2-fs time step for 20 nanoseconds (ns). During the equilibration process, we applied restraint potentials on the positions and dihedral angles of carbohydrate, lipid, hERG protein and rescue drug E-4031. The force constants for these restraints were progressively decreased before production. For the classical production run, each system was simulated for 200 nanoseconds (ns) using a 4-fs time step, employing the hydrogen mass repartitioning technique (Hopkins et al., 2015; Gao et al., 2021), and no restraint potentials were imposed. We applied the SHAKE algorithm to maintain bond lengths involving hydrogen atoms (Ryckaert et al., 1977). To handle interactions, we set a van der Waals interaction cutoff at 12 angstroms (Å) with a force-switching function operating between 10 and 12 Å^62^. Electrostatic interactions were computed using the particle mesh Ewald method (Darden et al., 1993). Temperature control was maintained at 310 K via Langevin dynamics (Farago, 2019) with a friction coefficient of 1 ps^−1^, while pressure was held at 1 bar using a semi-isotropic Monte Carlo barostat (Torrie and Valleau, 1977).

### 2.3 Accelerated molecular dynamics

Following the classical MD simulations, we employed Accelerated Molecular Dynamics (AMD) (Hamelberg et al., 2004; Grant et al., 2009; Bucher et al., 2011; de Oliveira et al., 2011; Mücksch and Urbassek, 2013; Gedeon et al., 2015) to enhance the exploration of conformational space for the wildtype and two mutant systems, as well as the two mutant systems in complex with the trafficking rescue drug E-4031. This approach aimed to reduce energy barriers within the simulation system. In this context, the simulation operates under the condition where the potential energy V(r) of the model system falls below a specified threshold energy level, denoted as E. When this condition is met, the simulation proceeds using a modified potential, denoted as V^*^(r) = V(r) + ΔV(r), where ΔV(r) represents a boost potential function defined as ΔV(r) = (E - V(r)) (Zequn and Jiangfang, 2021)/(α + (E - V(r))), where α is a parameter governing the degree of acceleration in the system (El-Sayed et al., 2022; Pawnikar et al., 2022).

To expedite all simulations, a dual boosting strategy was implemented, targeting both the total potential and dihedral potentials. The values of E and α were determined based on the average total potential energy and dihedral energy derived from 200 ns of unbiased MD simulation (as detailed in Supplementary Table S2). These boost potentials were subsequently applied to each system during a canonical AMD simulation conducted over 1.5 ms at 310 K in an explicit solvent environment, commencing from the final configuration obtained from the corresponding 200 ns unbiased MD simulation.

### 2.4 Clustering and analysis of MD simulations

To determine the dominant conformations of each protein, we utilized a clustering approach on the accelerated MD trajectory data. We conducted RMSD conformational clustering using the average-linkage algorithm, implemented in the cpptraj (Roe and Cheatham, 2013) utility of AMBER20 (Salomon-Ferrer et al., 2013). This involved a two-stage process for each simulation: initially, by exploring cluster counts ranging from 2 to 100, and subsequently, by employing a custom number of clusters specific to each system based on the outcomes of the first stage (refer to Supplementary Figure S1). It is worth noting that the use of the average-linkage algorithm in MD trajectories has been previously validated, distinguishing it among various clustering algorithms (Shao et al., 2007). Our specific focus in the clustering analysis was on the backbone atoms (Cα, C, N, and O) of the S6 helix within the transmembrane domain (TMD). The S6 helix constitutes one of the six helices within the transmembrane domain (Butler et al., 2020; Robertson and Morais-Cabral, 2020). Specifically, when four copies of the S6 helix converge—one from each channel monomer—they collectively form the channel pore. Notably, this helix accommodates all the residues for the rescue drug binding site. The choice of this helix is strategic, as it allows for the representation of a collective conformational change that considers all four monomers. During the course of simulations lasting 1.5 ms, snapshots were captured at intervals of 0.01 ns. To mitigate global translation and rotation effects, all backbone atoms were aligned using RMSD fitting to a reference conformation that had been reduced.

To evaluate the quality of the clustering results, we calculated two clustering metrics: the Davies-Bouldin index (DBI) and the “elbow criterion.” A high-quality clustering outcome is indicated When the Davies-Bouldin Index (DBI) attains a local minimum and the percentage of variance (SSR/SST) levels plateaus, it indicates the optimal number of clusters (Supplementary Figure S1). Conversely, the elbow criterion suggests that beyond the optimal number of clusters, there is a plateau in the fraction of variance explained by the data. Considering these factors, we chose to use 5 clusters for the simulations of the wildtype hERG, 5 clusters for the R534C mutant, and 3 clusters for the N470D mutant during the clustering process (Supplementary Figure S1).

For the analysis of MD trajectories, we employed the cpptraj software (Roe and Cheatham, 2013) and MDanalysis (Gowers et al., 2019) tools. We utilized the enhanced interface for the HOLE software (Smart et al., 1996) incorporated into the MDanalysis package (Gowers et al., 2019) to compute pore size. In contrast to the standalone HOLE software, the MDanalysis interface allows for the calculation of channel pore size as a function of MD simulation time. The Visualization of the simulations was conducted using VMD version 1.9.3 software (Humphrey et al., 1996), and the figures presented were generated using a combination of VMD (Humphrey et al., 1996) and the MOE software (Molecular Operating Environment MOE, 2022) packages.

## 3 Results and discussion

### 3.1 Building and simulating models of hERG wild-type and mutant variants

A model of the hERG channel wildtype tetramer was first constructed by utilizing the AlphaFold-generated hERG monomer model, as outlined in Section 2. Subsequently, to mimic the form of hERG found in the endoplasmic reticulum (ER), we introduced core glycosylation at residue N598. This core N-glycan consists of a precursor oligosaccharide group comprising 14 residues, including three glucoses, nine mannoses, and two N-acetyl glucosamine residues (Glc3Man9GlcNAc2). Using this wildtype model as a foundation, we proceeded to create two mutant models, N470D and R534C, along with another pair of mutant models complexed with the trafficking rescue drug E-4031. All five models were then embedded in a model of the ER membrane and subjected to simulations under physiological conditions for a duration of 1.5 μs

**FIGURE 2:**
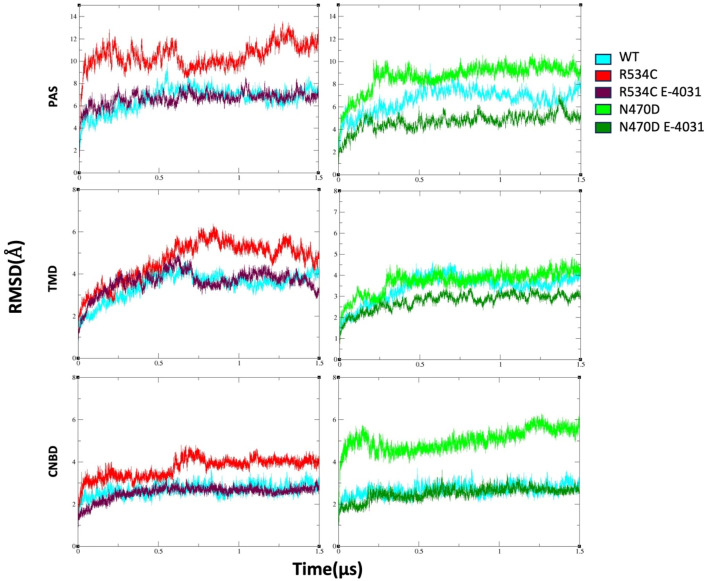
The backbone root-mean-square deviation (RMSD) of wildtype hERG and two hERG trafficking mutants with and without complexation with trafficking rescue drug E-4031. Calculation is made sepratly for each hERG domain, TMD, PAS, and CNBD.

In order to streamline our structural analysis and gain deeper insights while minimizing the impact of extensive loops in domain linkers, we conducted separate analyses for each domain of the channel and the calculations were averaged across the four copies of each domain in every simulation. After approximately 500 ns, the backbone root-mean-square deviation (RMSD) for the wildtype hERG converged to values of 3.5, 2.5, and 6 Å for its transmembrane domain (TMD), cyclic nucleotide-binding domain (CNBD), and Per-Arnt-Sim (PAS) domains, respectively, in comparison to the equilibrated structure (Figure 2). The backbone RMSD values were notably higher for the two hERG mutants across nearly all channel domains. Specifically, the PAS domain exhibited average RMSD values of 11 Å and 9 Å for mutants R534C and N470D, respectively. In the case of the TMD domain, the mutants R534C and N470D had average RMSD values of 5 Å and 4 Å, respectively, while for the CNBD domain, these values were 4 Å and 5.5 Å for mutants R534C and N470D, respectively. Also, to mitigate the inherent randomness of the MD simulations and ensure reproducibility, we repeated the classical portion of the MD simulations for the initial 200 ns, as illustrated in Supplementary Figures S2, S3. This approach enhances the robustness of our results.

However, for the mutants that were complexed with the trafficking rescue drug E-4031, the hERG channel displayed backbone RMSD values that were quite similar to those of the wildtype in all domains, except for the N470D-E4031 complex. The N470D-E4031 complex exhibited lower backbone RMSD values for both its PAS and TMD domains, with average values of 4.3 Å for the PAS domain and 2.8 Å for the TMD domain (Figure 2).

**FIGURE 3:**
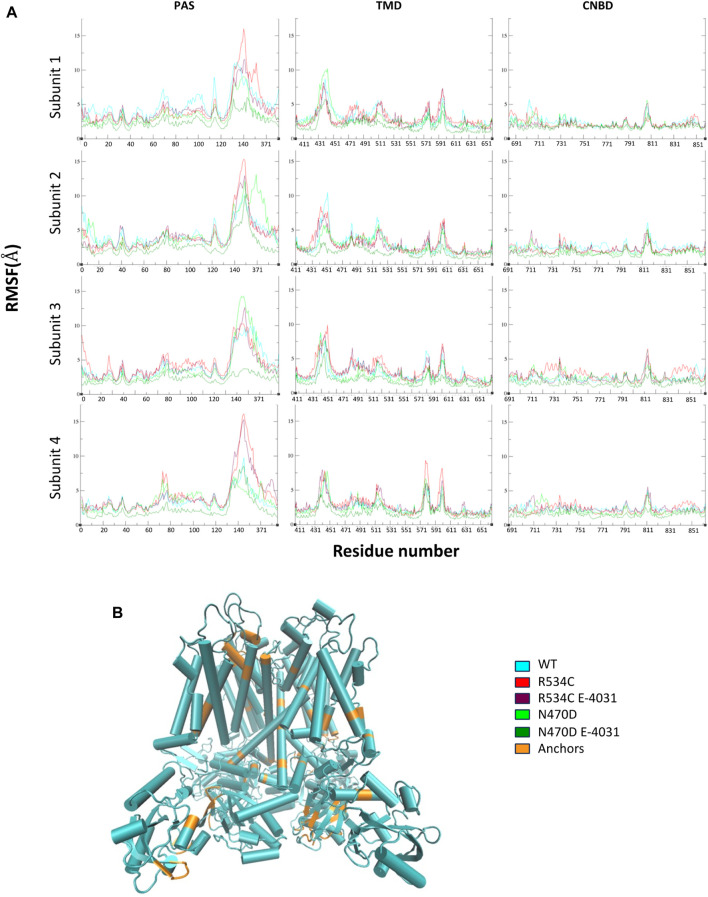
**(A)** Root mean square fluctuation of atomic positions (RMSF) of wildtype hERG and two hERG trafficking mutants with and without complexation with trafficking rescue drug E-4031, averaged over residues for all channel monomers, each hERG domain is displayed separately. **(B)** The tetrameric structure of the hERG channel highlighting the low RMSF segments that were used in the superposition of representative top clusters for each MD simulation.

To assess the mobility and dynamics of structural elements within the protein during all simulations, we examined the root mean square fluctuation of atomic positions (RMSF), which is averaged over residues for all channel monomers. As anticipated, we observed that several regions exhibited notably high RMSF values (Figure 3A). These regions correspond to amino acids found within the numerous loops present in each hERG domain (Figure 3B), as well as the inter-domain linkers, particularly those connecting the PAS domain to the TMD domain. Additionally, we noticed relatively lower RMSF values in the region of the CNBD domain. This observation can be attributed to the CNBD domain being tightly surrounded by the PAS domains of the channel monomers from all directions. These interactions at the interface of these two intracellular domains are of particular significance for regulating channel gating and functionality (Soohoo et al., 2022).

**FIGURE 4:**
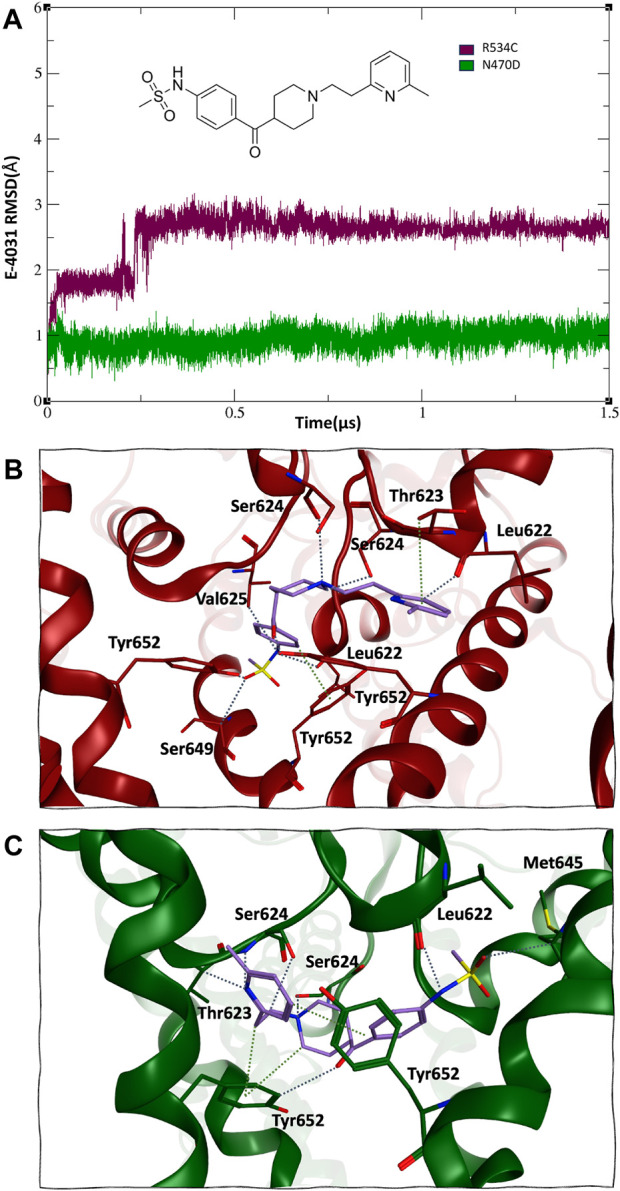
**(A)** 2D structure and RMSD of trafficking rescue drug E-4031 when bound to the two trafficking mutants of the hERG channel. **(B)** and **(C)**, mode of binding of trafficking rescue drug E-4031 inside the pore binding site of hERG mutant R534C and N470D respectively.

The binding with the rescue drug E-4031 was observed to have a moderating effect on the mobility of the channel protein across all domains of the channel, particularly in the case of the N470D-E4031 complex. This binding led to an enhancement in the overall structural stability of the entire channel. Notably, the N470D-E4031 mutant complex formed a robust interaction with the channel’s pore binding site throughout the entirety of the accelerated MD simulation, with an average ligand RMSD of only 1 Å from the docked pose. In contrast, the same ligand bound to mutant R534C exhibited convergence after 250 ns, with an average ligand RMSD of 2.7 Å from the docked pose, as shown in Figure 4A. Furthermore, we assessed the relative binding energies of the rescue drug E4031 simulated in the two hERG mutants using the Prime MM-GBSA module (Schrödinger Release, 2021) (refer to Supplementary Table S4). We selected the optimal docking poses of E-4031 after evaluating its alignment within the desired docking site in mutant models of the hERG channel to carry MD simulations.

Interestingly, despite both hERG mutations originating from the same docked pose within the hERG pore binding site (Wang and MacKinnon, 2017; Butler et al., 2020; Robertson and Morais-Cabral, 2020), the rescue drug E-4031 assumed a slightly different orientation. In the case of mutant R534C, the rescue drug engaged in hydrogen bonding interactions with multiple Tyr562 residues, as well as multiple Ser624 and Leu622 residues, with distances ranging from 2.7 to 2.9 Å. Additionally, its six-membered ring formed arene-hydrogen interactions with multiple Thr623 residues, as depicted in Figure 4B.

On the other hand, for mutant N470D, the trafficking rescue drug formed backbone hydrogen bonds with multiple Ser624 and Leu622 residues, with distances spanning from 2.9 to 3.3 Å. Furthermore, the drug engaged in arene-hydrogen interactions with the sidechain rings of several Tyr652 residues, as shown in Figure 4C.

### 3.2 Structural characteristics of the hERG pore domain

**FIGURE 5:**
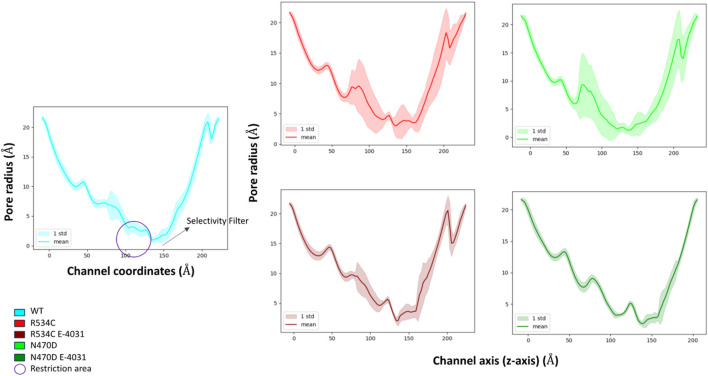
Pore radius analysis of wildtype hERG and two hERG trafficking mutants with and without complexation with trafficking rescue drug E-4031.

To assess the impact of rescuable mutations on the transmembrane domain, the common site of both mutations under examination, we conducted measurements of the hERG channel pore’s radius along the *z*-axis throughout the simulations for both the wildtype and mutant channels, as depicted in Figure 5. The channel pore radius serves as a comprehensive indicator of how the mutations affect the channel subunits in relation to one another.

The mean pore radius over the entire simulation trajectory provides valuable insights. It becomes evident that, in the case of the two mutants, N470D and R534C, as compared to the wildtype, the channel pore is undergoing a significant collapse, particularly in the region located just beneath the channel’s selectivity filter. This observation implies that the structural integrity of the entire channel pore is substantially compromised in both mutants.

Furthermore, when examining the standard deviations associated with the mean pore radius, it became apparent that the entire channel pore experienced substantial fluctuations in its structural stability in both mutant cases. This instability extended even to the selectivity filter, where the radius plays a crucial role in facilitating proper channel function and potassium selectivity.

Conversely, when these mutants form complexes with the trafficking rescue drug E-4031, the channel pore exhibits a mean radius that closely mirrors that of the wildtype hERG across the entirety of the channel coordinates. Additionally, it is noteworthy that binding to the rescue drug not only maintains but also stabilized the channel radius along its entire length, including the critical selectivity filter segment. Furthermore, the interaction of mutant N470D with the rescue drug E-4031 resulted in an additional level of stability for the channel pore structure when compared to the wildtype hERG.

To visually represent the destabilization observed in the channel pore across the simulated wildtype and mutant structures, we employed a superposition technique. Specifically, we superimposed the top clusters derived from clustering the three trajectories, utilizing the segments with the lowest root mean square fluctuation (RMSF) values as anchor points, as illustrated in Figure 3B.

**FIGURE 6:**
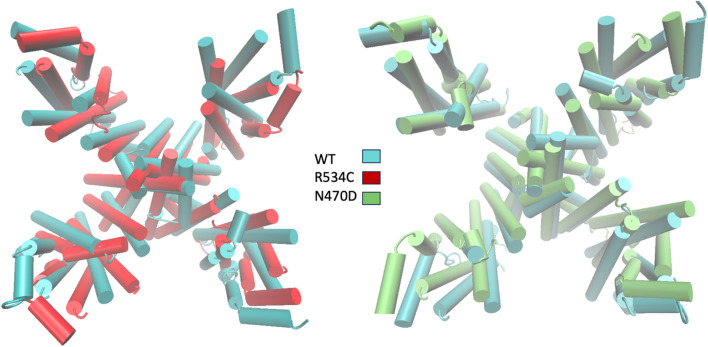
Superposition of representative top clusters from the accelerated MD simulation of transmembrane domain of the two hERG trafficking mutants on the representative structure of the hERG wildtype transmembrane domain. Superposition based on low RMSF anchor points.

In Figure 6, the transmembrane region of both wildtype and mutant channels is presented in a superimposed manner. The structures shown for each channel model—wildtype, mutant N470D, and mutant R534C hERG—depict the representative conformation of the most populated cluster obtained from the accelerated MD simulation of each model. This visualization highlights the constriction beneath the channel’s selectivity filter, demonstrating the complete closure of the S6 helices forming the pore in both mutants, N470D and R534C.

### 3.3 Structural characteristics of the hERG intracellular domains

The intracellular domains play a pivotal role in retaining structurally unstable hERG proteins within the endoplasmic reticulum (ER). Specifically, it is within the PAS and CNBD domains that various chaperone proteins responsible for aiding in the correct folding and tetramerization of the hERG protein bind. Additionally, these intracellular domains serve as points of interaction for ER quality control mechanisms, which communicate with improperly folded hERG proteins for subsequent degradation.

Our proposed hypothesis suggests that the structural destabilization observed in the transmembrane domain due to the rescuable trafficking mutations eventually extends to the intracellular domain. This, in turn, leads to structural disturbances within the intracellular domain itself, directly contributing to the retention of these mutant proteins within the ER.

**FIGURE 7:**
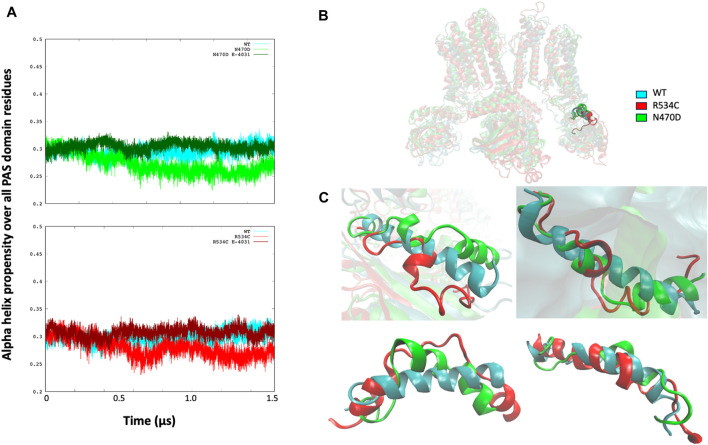
**(A)** Alpha helix propensity averaged over all PAS domain residues for wildtype hERG and two hERG trafficking mutants with and without complexation with trafficking rescue drug E-4031. **(B)** location of the alpha helix Lys362-Pro386 that is lost in both hERG mutants in relation to the PAS and TMD domains. **(C)** superposition of the alpha helix Lys362-Pro386 that is lost in both hERG mutants on the wildtype alpha helix for each channel monomer separately, structures of the alpha helices are from the representative conformation of the most populated cluster taken from clustering each simulation.

To examine the structural consequences of the transmembrane mutations on the two intracellular domains, we conducted an analysis of the secondary structure types over the course of the accelerated MD trajectories for the PAS and CNBD domains, as illustrated in Supplementary Figure S4. Our observations revealed that within the PAS domain of both mutants, N470D and R534C, there was a consistent decrease, averaging just over 5%, in the propensity for alpha-helical structures when compared to the wildtype simulation (refer to Figure 7A).

Upon closer examination of the PAS domain, we identified a specific alpha-helix within its linker region (Lys362-Pro386) that exhibited a nearly complete loss in all monomers of both mutant simulations, in stark contrast to the wildtype where it remained intact. This structural alteration is visually represented in Figure 7C, which presents a superposition of the Lys362-Pro386 alpha-helix from the representative top clusters of both the wildtype and mutant simulations. In this comparison, it becomes evident that the alpha-helix structure is severely disrupted in both the N470D and R534C mutants.

Regarding the mutants that formed complexes with the rescue drug E-4031, we observed that the propensity for the alpha helix secondary structure type closely resembled that of the wildtype throughout the entire accelerated MD simulation for both mutant complexes, as depicted in Figure 7A.

**FIGURE 8:**
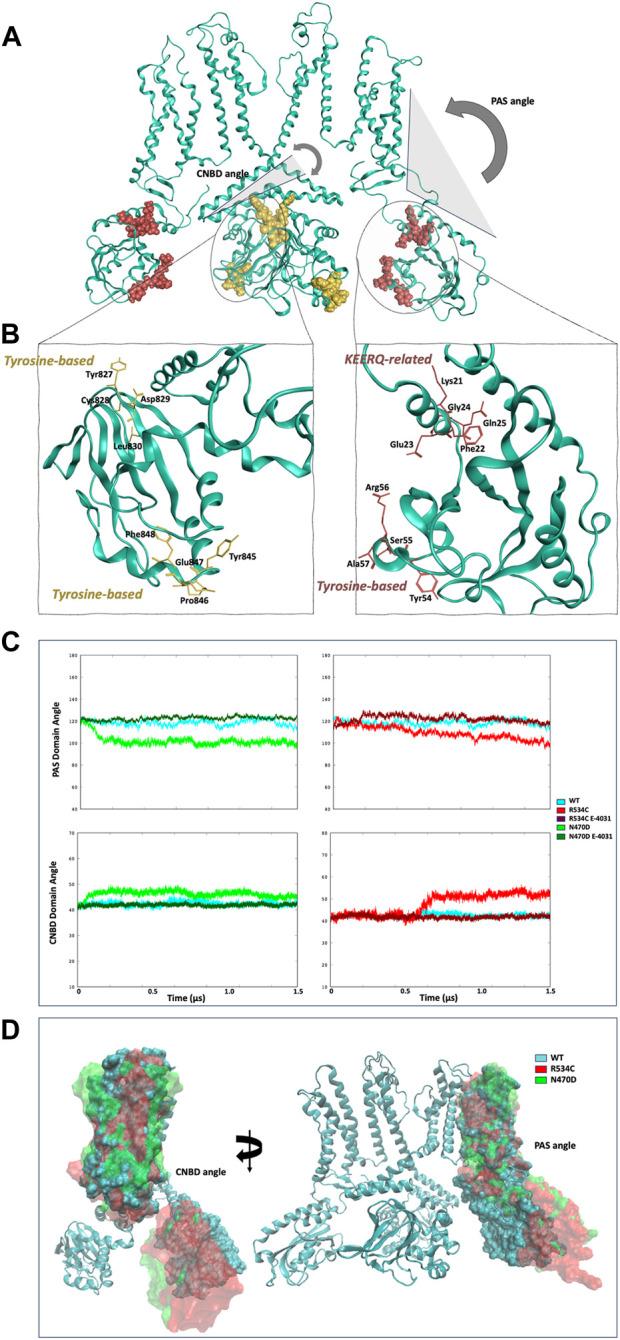
**(A)** illustration of the two angles that were calculated for each intracellular domain of the hERG channel. **(B)** internalization sequences found within the two intracellular domains of the hERG channel. **(C)** time series of the angles calculated for each of the two intracellular domains of the hERG channel. **(D)** visualization of the effect of the angle change on the conformation of both intracellular domains of the hERG channel.

We hypothesized that the absence of this specific alpha helix (as illustrated in Figure 7B) might have implications for the overall tertiary structure of the PAS domain in relation to the transmembrane domain. In order to investigate this, we computed the angle measurement between the center of mass of the PAS domain, the center of mass of the hinge segment connecting the PAS domain to the transmembrane domain, and the center of mass of the transmembrane domain, which we referred to as the “PAS domain angle.” A similar angle calculation was also performed for the other intracellular domain of hERG, the CNBD, and we termed this measurement the “CNBD domain angle.” (Figure 8A).

In the context of the PAS domain, our analysis revealed a reduction of slightly over 20° in its angle relative to the transmembrane domain (TMD) during the MD trajectories of mutants N470D and R534C when compared to the wildtype simulation, as presented in Figure 8C. This decrease in the angle suggests that the PAS domain in both hERG mutants gradually moves closer to the transmembrane domain as the simulation progresses. This conformational shift results in the exposure of specific linear internalization and lysosomal sorting motifs inherent in the protein sequence of the hERG PAS domain (Bonifacino and Traub, 2003; Dores et al., 2016).

It is important to emphasize that ER quality control mechanisms recognize hERG channels within the ER through the exposure of one or more of these sequences (Foo et al., 2019). In their research, Brian Foo and his team (Gedeon et al., 2015) identified three internalization signals in the PAS domain of the hERG channel and an additional three in the CNBD domain. Our investigation revealed that the conformational change we observed exposes two of these sorting signals within the PAS domain. Specifically, these signals include a KFERQ-related sequence (K21-Q25), associated with chaperone-mediated autophagy, and a tyrosine-based sequence (Y54-A57), which facilitates internalization and sorting to lysosomes by recruiting AP-2 and AP-3 clathrin adaptors (see Figure 8B).

In the case of the CNBD domain, we observed an increase of approximately 10° in the CNBD angle (as depicted in Figure 8C). This change indicates that in the conformational state adopted by the two trafficking-impaired mutants, the CNBD domain is moving away from the transmembrane domain. This shift in position leads to the exposure of two hERG internalization signals within the CNBD domain, specifically Y827-L830 and Y845-F848, both of which are tyrosine-based internalization sequences (as shown in Figure 8B).

Although the conformational alterations in these intracellular domains may seem modest, it is important to note that the structural destabilization within the rescuable hERG trafficking mutants is believed to be mild and reversible. Such changes can potentially account for the ER retention of these mutants.

**FIGURE 9:**
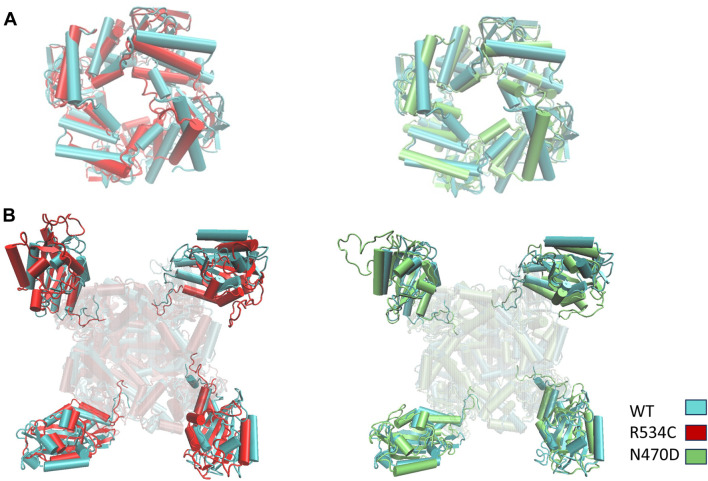
Superposition of representative top clusters from the accelerated MD simulation of the two intracellular domains **(A)** CNBD **(B)** PAS of the two hERG trafficking mutants on the representative structure of the hERG wildtype intracellular domains. Superposition based on low RMSF anchor points.

In the simulations of the two trafficking-defective mutants bound to the rescue drug E-4031, the angles calculated for both intracellular domains of the hERG channel remained consistent with values comparable to those of the wildtype throughout the entire accelerated MD simulations (Figure 8C). Also, Multiple replicas of classical molecular dynamics simulations for all five systems successfully reproduced the same angles between the PAS domains and the CNB domains, with deviations of approximately 5° from the original simulations as shown Supplementary Figure S5. Figure 8D illustrates how this angle change affects the structures of both the wildtype and the two mutants when we superimpose the transmembrane domain of the three representative conformations of each simulation, highlighting the impact of the angle change. Conversely, Figure 9 showcases the structural differences in the two intracellular domains of the mutants compared to the wildtype intracellular conformation when we base our superposition on anchor regions with low root mean square fluctuation (RMSF).

## 4 Conclusion

Our research has addressed a critical knowledge gap by focusing on dynamic computational models that encompass both the wildtype hERG voltage-gated potassium channel and trafficking-deficient mutants. We investigated the effect of two transmembrane domain located trafficking mutations R534C and N470D on the structure of the hERG protein and the influence of the trafficking rescue drug E-4031 on these mutants, shedding light on their structural dynamics.

To bridge this knowledge gap, we employed a comprehensive methodology involving modeling the complete channel protein and accelerated molecular dynamics simulations. Our results offer a microscopic understanding of the structural issues underlying the degradation of rescuable transmembrane trafficking mutations. These mutations induce conformational changes in the transmembrane domain, resulting in the narrowing of the main channel pore. This structural transformation extends to the two intracellular domains of the mutant hERG, leading to a conformational shift that exposes internalization signals. These signals likely contribute to the ER retention and subsequent degradation of rescuable, trafficking-deficient hERG channels. Our results also indicate that the rescue drug E-4031 exerts a stabilizing effect on the structure of trafficking mutants R534C and N470D preventing the structural destabilization of the channel protein.

Furthermore, it is important to note that hERG trafficking mutations exhibit diverse effects on intracellular transport and maturation, leading to various Long QT Syndrome (LQTS) symptoms. Importantly, not all hERG trafficking mutants can be rescued pharmacologically (Al-Moubarak et al., 2020; Luo et al., 2021; Oliveira-Mendes et al., 2021), and the effectiveness of rescue strategies varies depending on the specific protein domain in which the mutation is located. Mutations in the intracellular CNBD domain of hERG tend to resist pharmacological correction. This highlights the necessity of considering the mutation’s specific location when exploring potential rescue strategies, as not all mutants respond to the same drugs, and the mechanisms of misfolding and rescue may differ among trafficking mutants. In summary, our study provides a comprehensive understanding of these intricate structural and pharmacological aspects of hERG trafficking mutants (Ficker et al., 2000; Kupershmidt et al., 2002; Mesquita et al., 2019).

## Data Availability

The original contributions presented in the study are included in the article/Supplementary Material, further inquiries can be directed to the corresponding author.
