# Supplementary material for: Computational insights into the mechanisms underlying structural destabilization and recovery in trafficking-deficient hERG mutants
Source: Front Mol Biosci. 2024 Aug 13;11:1341727. doi: 10.3389/fmolb.2024.1341727 (PMC11347279; doi:10.3389/fmolb.2024.1341727)
Supplement: Supplementary file 1 [file DataSheet1.docx]

**Supporting Information**

**Computational Insights into the Mechanisms Underlying Structural Destabilization and Recovery in Trafficking-Deficient hERG Mutants**

Sara AlRawashdeh, Farag E.S. Mosa, Khaled H Barakat^*^

*Faculty of Pharmacy and Pharmaceutical Sciences, University of Alberta, Edmonton, AB, Canada.*

*Corresponding Author: [kbarakat@ualberta.ca](mailto:kbarakat@ualberta.ca)

**Table S1** Lipid composition of simulated endoplasmic reticulum membrane (ER) used in the simulations of hERG channels.

| Lipid Name | No. Of Lipids in Each Leaflet |
| --- | --- |
| POPC | 16 |
| PLPC | 18 |
| SAPC | 27 |
| POPE | 5 |
| PSPE | 8 |
| SAPE | 7 |
| SAPI | 3 |
| SLPI | 3 |
| OLPS | 3 |
| PSM | 4 |
| POPA | 1 |
| CHOL | 5 |
| TOTAL | **100** |

**Table S2** AMD parameters for boosting of total potential energy (α_tot_, E_tot_) and dihedral energy terms (α_dih_, E_dih_). Values in kcal/mol.

| Simulated System | α_tot_ | E_tot_ | α_dih_ | E_dih_ |
| --- | --- | --- | --- | --- |
| Wildtype | 174348.6 | -1594890.6 | 1825.6 | 65738 |
| R534C | 168462.8 | -1503312.7 | 1825.6 | 65845 |
| N470D | 168473.8 | -1503983.6 | 1825.6 | 65881 |
| R534C-E4031 | 168462.8 | -1503312.7 | 1825.6 | 65845 |
| N470D-E4031 | 168473.8 | -1503983.6 | 1825.6 | 65881 |

**Table S3** Rescue drug E-4031 GLIDE Docking Scores in the two mutant models of the hERG channel.

| **hERG Model** | **E-4031 GLIDE Docking Score (Kcal/Mol)** |
| --- | --- |
| **R534C** |  |
| **pose #1** | **-11.501** |
| **pose #2** | **-11.456** |
| **pose #3** | **-11.260** |
| **pose #4** | **-11.078** |
| **pose #5** | **-11.061** |
| **N470D** |  |
| **pose #1** | **-11.857** |
| **pose #2** | **-10.139** |
| **pose #3** | **-9.907** |
| **pose #4** | **-9.703** |
| **pose #5** | **-9.643** |

**Table S4** Rescue drug E-4031 Prime MM-GBSA Scores in the two mutant models of the hERG channel.

| **hERG Model** | **MM/GBSA score (Kcal/Mol)** |
| --- | --- |
| **R534C** |  |
| **pose #1** | **-83.72** |
| **pose #2** | **-81.57** |
| **pose #3** | **-81.10** |
| **pose #4** | **-80.11** |
| **pose #5** | **-75.50** |
| **N470D** |  |
| **pose #1** | **-93.05** |
| **pose #2** | **-81.64** |
| **pose #3** | **-54.47** |
| **pose #4** | **-35.69** |
| **pose #5** | **-24.08** |

**Figure S1** RMSD based Conformational clustering cluster metrics of the S6 pore helix in the wildtype and hERG trafficking mutants using 1500ns MD trajectories.


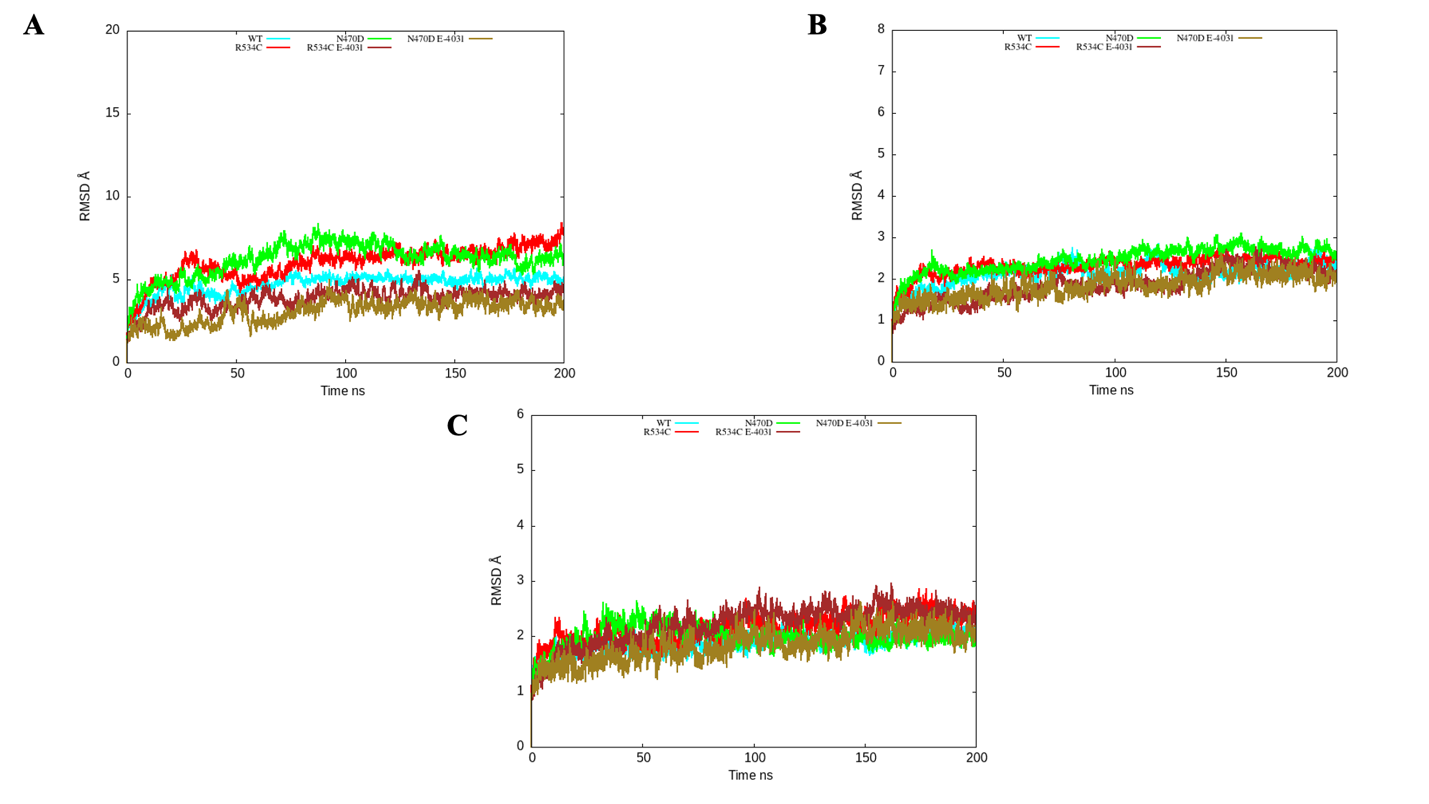


***Figure S2*** *Depicts the backbone root-mean-square deviation (RMSD) of wild-type hERG and two hERG trafficking mutants, both with and without complexation with the trafficking rescue drug E-4031. The calculations are performed separately for each hERG domain: A) PAS domain TMD, B) transmembrane domain (TMD), and C) cyclic nucleotide-binding domain (CNBD).*


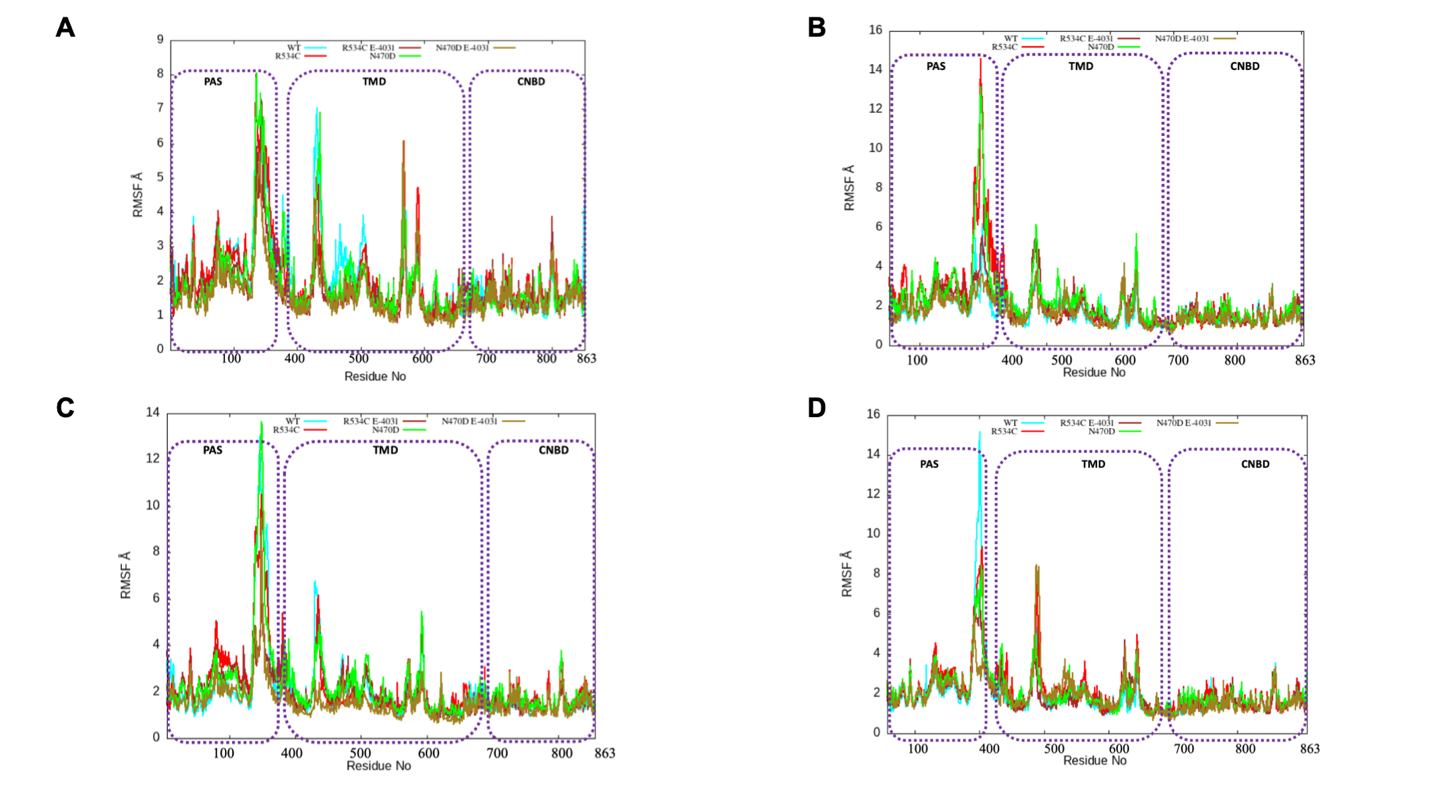


***Figure S3*** *Root Mean Square Fluctuation (RMSF) profiles were analyzed for the wild-type hERG and two hERG trafficking mutants, both with and without complexation with the trafficking rescue drug E-4031 and each domain highlighted.*


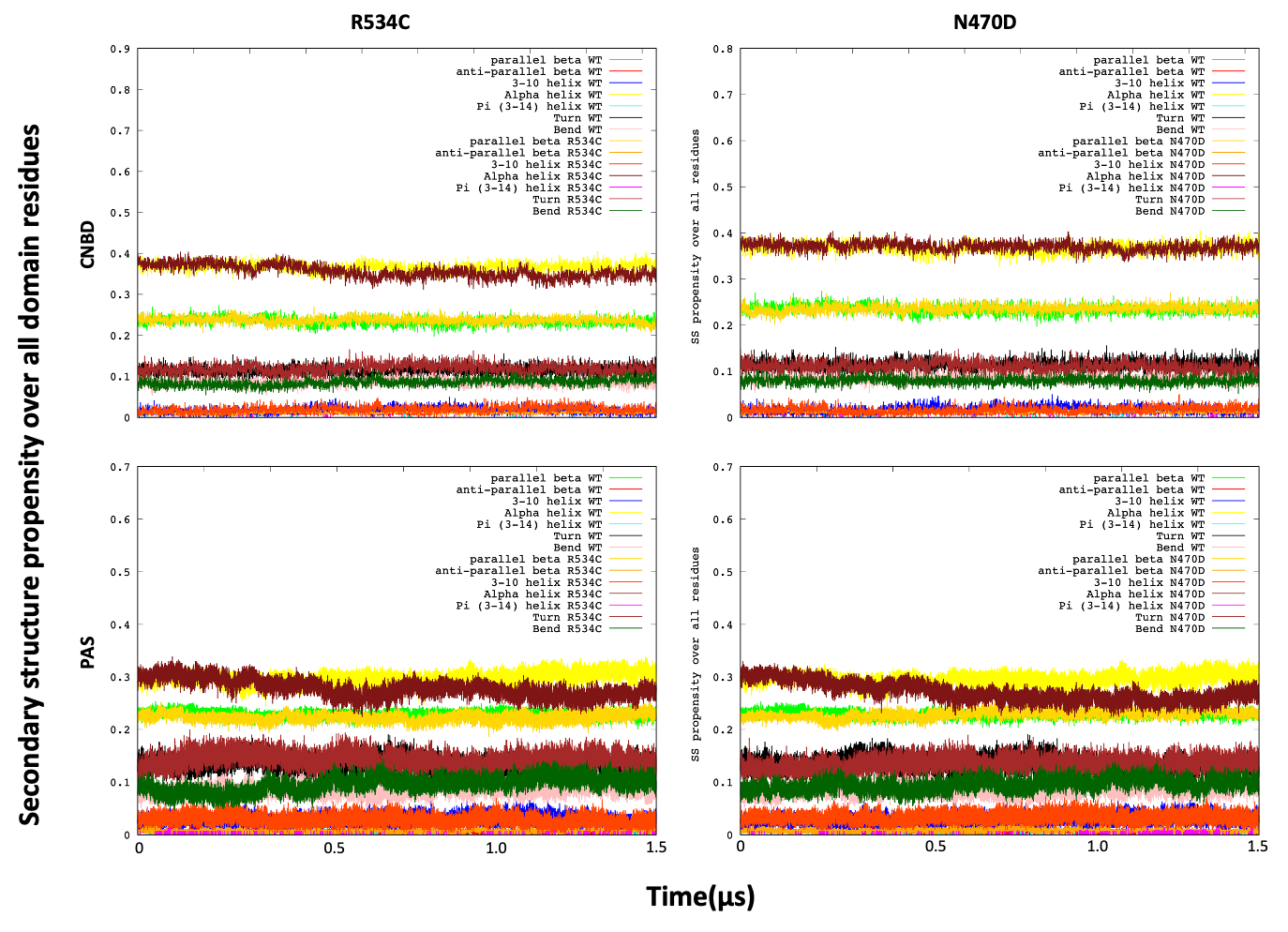


**Figure S4** Analysis of the secondary structure types propensities over the course of 1500ns of the accelerated MD trajectories for the PAS and CNBD domain in the wildtype and two traffecking mutants of the hERG channel.


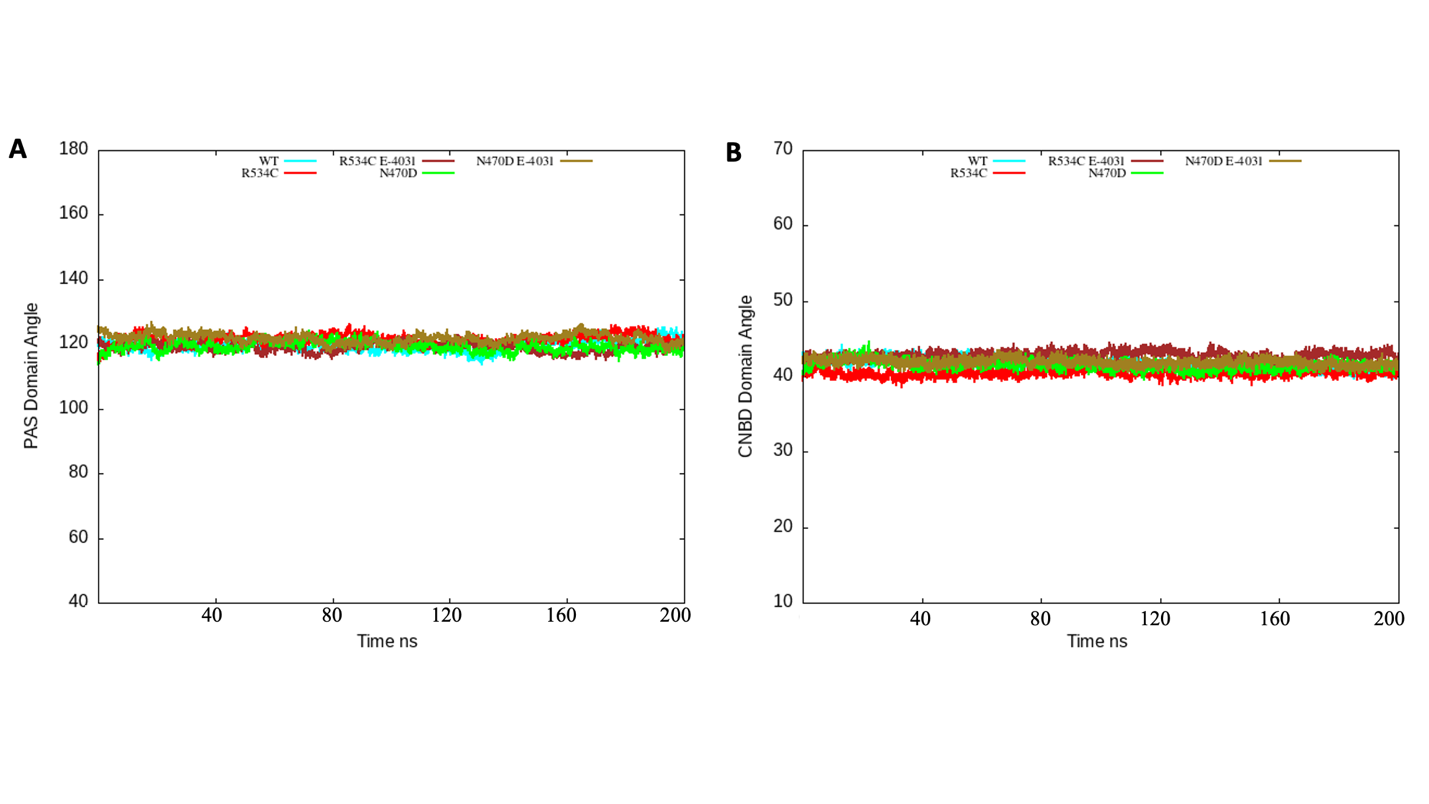
 ***Figure S5*** *Time series analysis of the angles measured for each of the two intracellular domains of the hERG channel over 200 ns classic MD simulation.*
